# PRMT6 mediates inflammation via activation of the NF-κB/p65 pathway on a cigarette smoke extract-induced murine emphysema model

**DOI:** 10.18332/tid/116413

**Published:** 2020-02-04

**Authors:** Xue He, Tiao Li, Lijuan Luo, Huihui Zeng, Yan Chen, Shan Cai

**Affiliations:** 1 Department of Pulmonary and Critical Care Medicine, The Second Xiangya Hospital, Central South University, Changsha, China; 2 Research Unit of Respiratory Disease, Central South University, Changsha, China; 3 Diagnosis and Treatment Center of Respiratory Disease, Central South University, Changsha, China

**Keywords:** chronic obstructive pulmonary disease, cigarette smoke extract, inflammation, nuclear factor-𝜅B, H3K4me3

## Abstract

**INTRODUCTION:**

Smoke-driven lung inflammation is considered to be the major pathophysiology mechanism of Chronic Obstructive Pulmonary Disease (COPD)/emphysema. Protein arginine methyltransferase 6 (PRMT6) is a key epigenetic enzyme, which is related to protecting the tri-methylation of H3K4 (H3K4me3). We hypothesized that PTMT6 protects lung inflammation through the nuclear factor kappa B (NF-κB) pathway.

**METHODS:**

Mice were injected with cigarette smoke extract (CSE) or PBS to establish a mice model, intratracheally instilled with overexpressed PRMT6 or negative control vector. Morphometry of lung slides and lung function were measured. We determined the protein expression of PRMT6 and its related histone targets, the activation of NF-κB pathway, the level of tumor necrosis factor α (TNFα) and interleukin-1β (IL-1β).

**RESULTS:**

After PRMT6 overexpression, the morphometry indexes and lung function were improved. Also, the expression of H3K4me3 was decreased. Overexpressed PRMT6 could suppress CSE-induced NF-κB activation and pro-inflammation genes expression.

**CONCLUSIONS:**

The overexpressed PRMT6 could serve as an inflammation inhibitor, potentially through blocking the NF-κB/p65 pathway in the murine emphysema model.

## INTRODUCTION

Chronic Obstructive Pulmonary Disease (COPD) is a growing public health concern, accounting for 6% of all deaths globally in 2012^1^. According to the definition in the Global Initiative for Chronic Obstructive Lung Disease (GOLD), COPD is regarded as a preventable and treatable chronic airway disease, which is characterized by persistent respiratory symptoms and airflow limitation^1^. The most common risk factor responsible for COPD is significant exposure to noxious particles or gases, especially cigarette smoke (CS), which leads to airway and/or alveolar abnormalities^1,2^. CS-induced chronic inflammation causes structural changes including the destruction of the lung parenchyma and narrowing of the small airways^1,3^. CS exposure is considered to be the major driver of emphysema^1^.

The nuclear factor kappa B (NF-κB) is one of the most important transcriptional factors that plays a major role in inflammatory lung diseases like COPD and asthma^4-6^. NF-κB is required for the transcription of many inflammatory genes involved in lung diseases, including interleukin-1β (IL-1β), IL-6, and tumor necrosis factor α (TNFα). NF-κB is located in the cytoplasm of normal cells, but it could migrate to the nucleus to induce genes expression under stimuli such as smoking and lipopolysaccharide (LPS)^6^.

Protein arginine methyltransferases (PRMTs) mainly catalyze arginine methylation of chromatin histones. There are nine PRMTs, which are categorized as four types by different catalytic products^7^. PRMT6 is the type I PRMT, which catalyzes asymmetric demethylation as well as PRMT1–4 and PRMT8. It has been reported that PRMT1 and 4 are considered to be correlated with NF-κB-dependent gene expression^8,9^. PRMT6 catalyzes the methylation of histone h3 arginine 2 (H3R2me2a). The methylations of H3 lysine 4 (H3K4me2, H3K4me3) and H3R2me2a appear to counter-correlate in the E-box-containing gene promoters^10,11^. Interestingly, H3K4me3, as a promoter marker, is correlated with gene expression^12-15^. The H3K4 tri-methylations synergistically regulate the activation of the NF-κB signaling pathway^16,17^. Although PRMT6 is responsible for endothelial inflammation induced by cigarette smoke extract (CSE), as shown in our previous study, its role and possible pathway have remained unclear.

In the present study, we examined whether PRMT6 could attenuate the inflammation of COPD and investigated the relationship between PRMT6 and NF-κB signaling pathway in a CSE-induced murine emphysema model. Our data indicate that the activation of NF-κB was negatively regulated by PRMT6 in the emphysema model, which was probably induced by tri-methylation of H3K4.

## METHODS

### Preparation of CSE

Briefly, in accordance with the method proposed by Zhang et al.^18^, a cigarette (Furong, China Tobacco Hunan Industrial Company; tar: 12 mg, nicotine: 1.1 mg, carbon monoxide: 14 mg) was burned and collected in a vessel containing phosphate-buffered saline (PBS: 2 mL) using a vacuum pump. The pH of the solution was adjusted to 7.2–7.4 and the solution was then passed through a microfilter with a pore size of 0.22 μM. CSE was prepared fresh before each use.

### Lentiviral particles

The lentivirus containing the protein arginine N-methyltransferase 6 gene and green fluorescent protein (GFP) was previously prepared, which was purchased from Invitrogen Trading (Shanghai) Co., Ltd, as well as the negative control lentivirus. The lentivirus titer was 1.0×10^9^ ifu/mL. The lentivirus was aliquoted and underwent no more than three freeze-thaw cycles. All the lentivirus was stored at -80 °C for no more than six months.

### Animal protocols

The animal care and experimental protocols were approved by the Animal Care and Use Committee of the Second Xiangya Hospital, Central South University. Six-week-old male specific-pathogen-free BALB/c mice (21–23 g each) were randomly divided into four groups: the control group, CSE group, CSE+Lenti-NC group, and CSE+Lenti-PRMT6 group. There were 8 mice per group. The control group and the other three groups were injected intraperitoneally with 0.3 mL PBS or CSE at days 0, 11, and 22, respectively^18,19^. The control group and CSE group received intratracheal instillation of PBS at day 14. The CSE+Lenti-NC group and CSE+Lenti-PRMT6 group received an intratracheal instillation of 108 ifu negative control lentivirus or PRMT6 overexpression lentivirus at day 14, respectively. On day 28, all mice were killed and lung tissues were obtained as described below.

### Lung function

On the 28th day, the extremities of mice were fixed and the neck was shaved after the anesthesia. After disinfection, the skin was cut off and the trachea was exposed bluntly. Damage to the surrounding blood vessels was avoided, a 22G intravenous catheter needle in the tracheal cartilage ring at the level of the anterior tracheal wall was introduced and connected to the plethysmograph chamber (Buxco Respiratory Products, Wilmington, NC, USA). The mice were allowed to breathe quietly. The respiratory frequency (f), tidal volume (TV), airway resistance (Raw) and dynamic lung compliance (Cdyn) of each group were recorded. All operations were performed by the same teacher at the Innovative Experimental Platform of Xiangya Medical College of Central South University to minimize the impact on the results and to ensure the accuracy of the operation and results. The teacher’s operation was undertaken in a blinded manner.

### Lung tissues preparation

The left main bronchus was ligated and 4% paraformaldehyde was infused into the left lung at a pressure of 25 cmH2O to inflate it. After that, it was fixed in 4% paraformaldehyde for at least 24 hours followed by paraffin-embedded sections, which were prepared using standard techniques. The right lung was collected in the cryopreservation tube and stored in liquid nitrogen for Western blotting analysis.

### Morphometry

The paraffin sections of lung tissue were stained with hematoxylin and eosin (HE). The mean linear intercept (MLI) and destructive index (DI) were measured to assess emphysema of lung tissue as previously described^18,19^. The preparation of the murine lung histopathology specimen and morphological analysis were performed in a professional pathology laboratory.

### Immunohistochemistry for localization and expression of NF-κB/p65

Immunohistochemistry was performed on lung sections to detect the expression and localization of NF-κB/p65. After deparaffinizing and rehydration, the primary antibody was incubated with anti-p65 (10745-1-AP, Proteintech, USA) at 4 °C overnight. Slides were incubated with biotin-conjugated secondary antibody and horseradish peroxidase-conjugated streptavidin. PBS was used to take the place of the primary antibody in negative control slides. Diaminobenzidine (DAB) was added and re-dyed. The results were evaluated by the percentage of nuclear positive cells in fields under ×400 magnification and pictures taken.

### Western blotting

Lung tissues were lysed on ice for 20 minutes in RIPA lysis buffer and then homogenized. The BCA protein-quantification kit (Beyotime, China) was used to measure the protein concentration. The total protein was separated on SDS-PAGE gel (Beyotime, China) and transferred onto the PVDF membranes (Millipore, USA). After blocked for one and a half hours by 5% non-fat dry milk and then washed, these membranes were incubated overnight with rabbit polyclonal antibodies at 4°C. The antibodies were: PRMT6 #14641S and H3K4me3 #9727S, (Cell Signaling Technology, USA); H3R2me2a ab8046, (Abcam, USA); TNF-α Sc-8301 and IL-1β Sc-7884 (Santa Cruz Biotechnology, USA). These membranes were incubated with HRP-labeled goat anti-rabbit secondary antibody (Cell Signaling Technology, USA) on the next day. Labeled proteins were visualized by the ECL plus Western blotting detection system (Bio-Rad, USA). Band densities were quantified using ImageJ software.

### Statistical analysis

Software packages (SPSS 19.0, SPSS Inc., USA & GraphPad Prism 8, USA) were used to perform all statistical analyses. The Shapiro-Wilk test was used to test the data distribution. Continuous data were expressed as mean ± standard deviation (SD). Differences were evaluated by the unpaired Student’s t-test for multiple comparisons. The correlations between NF-κB and the expression of H3K4me3 in mice was tested by Pearson correlation. A p-value <0.05 was considered statistically significant.

## RESULTS

### Lung function

**Figure 1:**
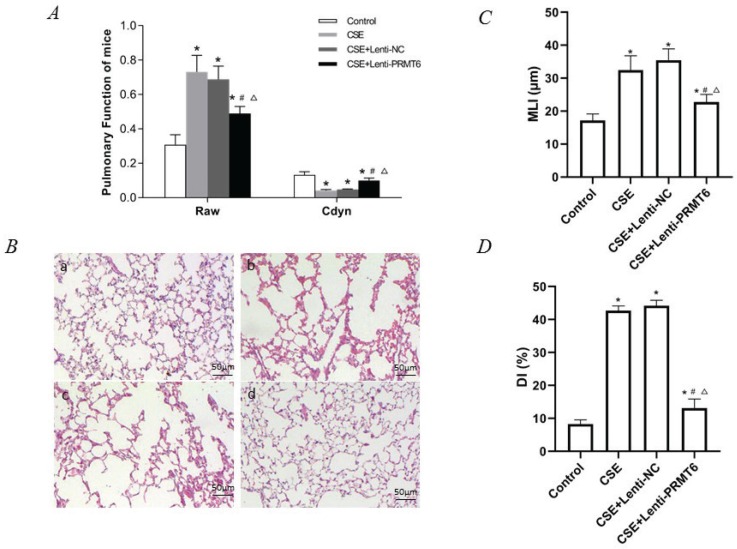
Lung function and histological examination of mouse lungs. (A) Raw and Cdyn were recovered after PRMT6 treatment; n=8 mice per group. (B) HE staining of lung slides (×200) in the control group (a), CSE group (b), CSE+Lenti-NC group (c), and CSE+Lenti-PRMT6 group (d); n=4 mice per group. MLI (C) and DI (D) were calculated in each group. *p<0.05 in comparison to control group; ^#^p<0.05 in comparison to CSE group; Δp<0.05 in comparison to CSE+Lenti-NC group

Usually, this mice model is known as an emphysema model after CSE stimulation. However, we surprisingly found that there is abnormal airflow in this model. The F, TV, RAW, and Cdyn, which represent the resistance of small airway and elasticity, were measured before the mice were sacrificed. As shown in Table 1 and Figure 1A, compared with the control group, RAW in the other three groups was significantly increased, Cdyn was significantly decreased, and the difference was statistically significant (p<0.05). This means that there is obstructed airflow in the mice model. Moreover, treatment with PRMT6 via the airway significantly protects the lung function of the mice.

**Table 1:** Lung function (Mean±SD)

*Parameters*	*Control group*	*CSE group*	*CSE+Lenti-NC group*	*CSE+Lenti-PRMT6 group*
F (breaths/min)	183±16.24	186±17.98	190±15.32	185±13.77
TV (mL)	0.22±0.011	0.23±0.012	0.23±0.019	0.21±0.013
RAW (cmH_2_O/mL/min)	0.329±0.058	0.732±0.096*	0.618±0.077*	0.470±0.041*^#^^Δ^
Cdyn (mL/cmH_2_O)	0.123±0.018	0.042±0.005*	0.048±0.004*	0.100±0.014*^#^^Δ^

* p<0.05, in comparison to the control group.

# p<0.05, in comparison to the CSE group.

Δ p<0.05, in comparison to the CSE + Lenti-NC group.

### Histological examination of mice

Morphometry of the lung gives direct evidence of emphysema. HE staining of lung tissue slices was analyzed at a magnification of ×100. It can be seen that the control group presented generally normal lung morphology without emphysema (Figure 1B-a). In the CSE group and CSE+Lenti-NC group, the alveolar space was increased and the alveolar wall was destroyed, showing the fusion of alveolar (Figure 1B-b, -c). However, there was only mild alveolar enlargement and less alveolar wall destruction after PRMT6 treatment (Figure 1B-d).

The morphological quantitative analysis of the pathological lung tissue sections of mice showed that compared with the control group, the MLI and DI in the other three groups were significantly increased (p<0.05), compared with the CSE group and CSE + Lenti-NC group. After the instillation of PRMT6, the morphology measures were partly recovered (p<0.05, Figures 1C, 1D).

### Expression of PRMT6, H3R2me2a, and H3K4me3 in mice

**Figure 2:**
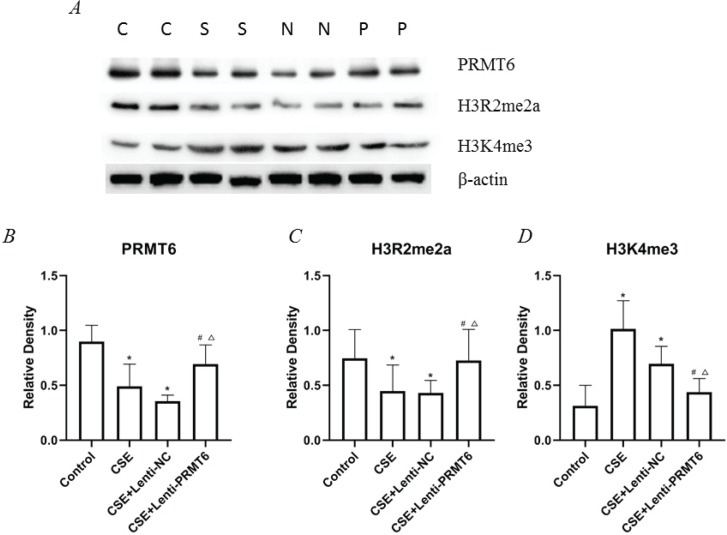
Expression of epigenetic protein in mouse lung. (A) Western blot analysis showed that overexpressing of PRMT6 caused significant changes of downstream target; n=4 mice per group. C: control group, S: CSE group, N: CSE+Lenti-NC group, P: CSE+Lenti-PRMT6 group. Relative density of PRMT6 (B), H3R2me2a (C) and H3K4me3 (D) were performed. *p<0.05 in comparison to control group; ^#^p<0.05 in comparison to CSE group; Δp<0.05 in comparison to CSE+Lenti-NC group

We determined the protein expression of PRMT6, H3R2me2a, and H3K4me3 (shown in Figure 2). Obviously, CSE injection impeded the PRMT6 protein level, which means there should be some biologic functions blocked. As the direct target of PRMT6, the expression of H3R2me2a was decreased. Subsequently, the amount of H3K4me3 was upregulated. For the PRMT6-overexpressed mice, this protein axis was rescued, surprisingly.

### Expression of NF-κB/p65 in mice

**Figure 3:**
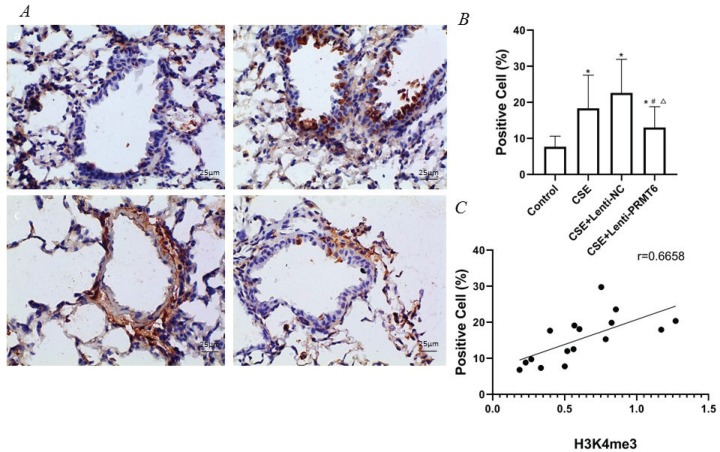
Immunohistochemical detection of NF-κB/p65 molecule and its correlation with H3K4me3. (A) Activated NF-κB/p65 were transferred into the nucleus (×400); n=4 mice per group. Dark brown nuclear cells were positive cells, shown in the control group (a), CSE group (b), CSE+Lenti-NC group (c), and CSE+Lenti-PRMT6 group (d). The percentage of positive cells (B) and its correlation with H3K4me3 (C) were performed. *p<0.05 in comparison to control group; ^#^p<0.05 in comparison to CSE group; ^Δ^p<0.05 in comparison to CSE+Lenti-NC group

Activated NF-κB/p65 transferred into the nucleus, regulates gene transcription and expression. The dark brown nuclear positive cells using immunohistochemistry represented activated NF-κB/p65. The slides showed that the activated NF-κB/p65 in the CSE group (Figure 3A-b) was significantly increased compared with the control group (Figure 3A-a). After overexpression of PRMT6, the nuclear translocation NF-κB/p65 was significantly reduced (Figure 3A-d). Linear regression analysis showed that the percentage of nuclear positive cells was positively correlated with H3K4me3 (Figure 3C, r=0.6658, p<0.05).

### Effect of PRMT6 on inflammation in the lungs of mice with emphysema induced by CSE

**Figure 4:**
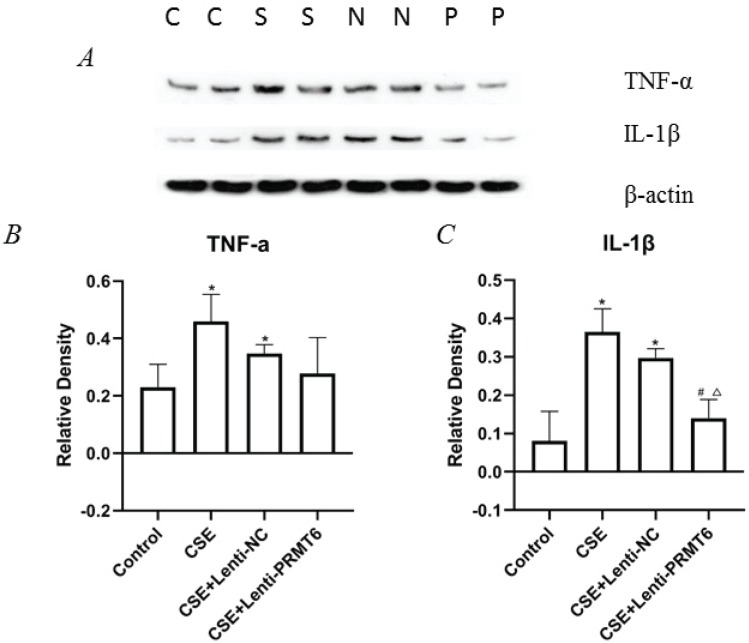
Effect of PRMT6 on inflammation in mouse lung. (A) Western blot analysis showed that overexpression of PRMT6 decreased pro-inflammatory factors; n=4 mice per group. Relative density of TNF-α (B) and IL-1β (C) were performed. *p<0.05 in comparison to control group; ^#^p<0.05 in comparison to CSE group; ^Δ^p<0.05 in comparison to CSE+Lenti-NC group

To examine whether the emphysema-protective effect of PRMT6 was associated with an anti-inflammatory effect on the mice after CSE injection, we measured the levels of TNFα and IL-1β in the lung tissues. NF-κB is a mediator of pro-inflammatory gene induction including TNFα and IL-1β. As shown in Figure 4, the levels of TNFα and IL-1β were significantly increased in the CSE group compared with those in the controls. Overexpression of PRMT6 treatment significantly reduced the levels of IL-1β, pro-inflammatory factor, in the lungs of CSE-treated emphysematous mice. The expression of TNFα was also decreased, although there was no significant difference.

## DISCUSSION

The present study confirmed the findings that PRMT6, an essential epigenetic enzyme, contributed to the beneficial effects on CSE-induced murine emphysema. Overexpressed PRMT6 could repress the activation of the NF-κB pathway and then block the release of pro-inflammatory cytokine levels (such as TNFα and IL-1β) in vivo. Moreover, the underlying mechanism may be involved in the tri-methylation of H3K4 in lung tissues of mice.

We demonstrated a novel and time-saving method to establish the murine model as previously published^18,19^. The most convenient phenotype was emphysematous lung tissue including destroyed alveolar wall and fusion of alveolar. The most highlighted finding was that the pulmonary function showed that the resistance of the airway was significantly higher in emphysema mice. In addition, Cdyn, not Static compliance (Cstat), was significantly decreased. It is well known that Cdyn represents pulmonary compliance during periods of gas flow, such as during active inspiration. So, Cdyn is used to underestimate the function of small airways in COPD/emphysema. When there was obstruction in the small airways, the airway resistance increased and compliance during inspiration decreased. It demonstrated that there was not only the existence of emphysema phenotype but also airflow limitation. However, the damaged lung function was improved after the instillation of PRMT6. Yu et al.^20^ used a demethylase inhibitor, which could modulate cell function by targeting H3K27 in airway smooth muscle cells, to treat asthma mice. They found that both lung-resistance and Cdyn were improved, and this inhibitor could attenuate airway remodeling via Akt/MAPKs signaling pathway. This analysis found evidence that methylation of histone h3 in the murine lung has some potential effect on lung structure and lung function. Further investigation is needed to explore the mechanism of damaged lung function in the CSE-injected-intraperitoneally induced COPD/emphysema murine model.

In recent decades, epigenetics has opened a new research avenue for pathogenesis mechanisms of COPD^21^. It is noteworthy that the key risk factor of COPD is cigarette smoking1. CS-related epigenetic modification has been reported^21^ that contributes to DNA methylation, histone methylation, histone acetylation etc. These modifications could affect thousands of gene expressions without DNA sequence changes. Take histone acetylation, for example, histone deacetylases (HDACs) have been well known as negative regulators of inflammation pathways in COPD and could act as potential therapeutic targets of COPD^22,23^. There is less research on histone methylation in COPD compared with acetylation. The methylation of histone h3 lysine 27 and lysine 4 have been published in CS-related experiments^21,24^. In our previous study, the tri-methylation of H3K4 was found to be related to apoptosis and oxidative stress in a murine emphysema model, as well as the expression of inflammation genes in lung tissue^25^. But it remains unclear how histone methylation accounts for lung inflammation in COPD.

PRMTs are a family of widespread enzymes. In this current study, we found that PRMT6 could act as a therapeutic protein in COPD. PRMT6 is known as a specific H3R2-methylating enzyme both in vivo and in vitro^10,11^. In most cases, H3K4me3 is recognized to mark the promoter regions of active transcription^15^. Thus, this is one of the possible reasons that H3K4me3 is related to p65 gene transcription. On the other hand, Aimee et al.^26^ found that H3R2 methylation by PRMT6 would detach K4me3-binding proteins and their associated transcriptional coactivators. Song et al.^17^ demonstrated that H3K4 tri-methyltransferase ASH2 could modulate to a certain extent pro-inflammatory transcription, such as TNF-α, by altering the affinity of p65 for target promoters. Transcription activation is coupled to the accumulation of active histone marks (such as H3K4me3). Similarly, Yu et al.^27^ indicated that H3K4 tri-methyltransferase SET1 could be recruited to activate the transcription of pro-inflammatory cytokines relying on NF-κB. We might infer that PRMT6 could reduce the level of H3K4 tri-methylation and block pro-inflammatory genes expression.

### Limitations

The main limitation of our current study is that we did not evaluate the binding between H3K4me3 and NF-κB/p65 signaling pathway. Our results may not be sufficient to conclude that PRMT6 would activate NF-κB directly. Further experiments in vitro are needed to investigate the potential mechanism of PRMT6 and lung inflammation in COPD.

## CONCLUSIONS

Chronic inflammation is a fundamental pathophysiology mechanism of COPD. It is surprising to observe that the overexpressed PRMT6 could serve as an inflammation inhibitor by blocking the NF-κB/p65 pathway in the murine emphysema model. It is a remarkable finding that provides a new therapeutic target for COPD.
